# Adolescent help-seeking on a psychological support hotline: latent classes, predictors, and distal outcomes

**DOI:** 10.3389/fpsyt.2025.1723286

**Published:** 2026-01-05

**Authors:** Zhihua Xie, Yanhong Hu, Yueshi Ye, Yongsheng Li, Lei Li, Maozeng Yang, Zezhi Li

**Affiliations:** 1 Department of Emergency Medicine, Dongguan Seventh People’s Hospital, Dongguan, China; 2 Department of Clinical Psychology, Dongguan Seventh People’s Hospital, Dongguan, China; 3 Office of the Director, Dongguan Seventh People’s Hospital, Dongguan, China; 4 Department of Medical Administration, Dongguan Seventh People’s Hospital, Dongguan, China; 5 Department of Nutritional and Metabolic Psychiatry, Affiliated Brain Hospital of Guangzhou Medical University, Guangzhou, China

**Keywords:** adolescent, hotline, help-seeking, latent class analysis, suicidal ideation, mixture models

## Abstract

**Background:**

Adolescents who contact psychological hotlines present with diverse problems and levels of risk. Recognizing common patterns quickly can help counselors match responses to need. This study aimed to identify distinct profiles of adolescent help-seeking and examine how those profiles relate to caller characteristics and near-term outcomes.

**Methods:**

We analyzed de-identified records from a municipal hotline in Dongguan, China (January 2020–December 2024). The analytic cohort included 2,300 callers aged 10–19 years. Latent class analysis used routinely recorded indicators: presenting problems (family, non-family interpersonal/peer, study/academic) and counselor ratings of hope, psychological pain, and wish-to-die/suicidal ideation (treated as ordered categories). Model selection balanced information criteria, likelihood-ratio tests, entropy, and interpretability. Misclassification corrected three-step models related caller and call characteristics to class membership, and distal outcomes included near-term suicidal-ideation intensity, 30-day re-contact, and referral/triage.

**Results:**

A four-class solution best balanced fit and parsimony. *Academic Strain* (39.2%) featured academic concerns with comparatively high hope and low immediate risk. *Family Conflict* (28.8%) showed moderate distress and intermediate risk. *Peer Conflict* (22.1%) was marked by higher psychological pain and elevated ideation. *Acute Suicidal Crisis* (9.9%) combined low hope, severe pain, and frequent active ideation. Females had higher odds of *Family Conflict* and *Acute Suicidal Crisis*; later school stages were associated with *Peer Conflict*. Prior psychiatric diagnosis, past self-harm, and longer calls distinguished the crisis class. Distal outcomes followed a clear gradient: mean near-term suicidal-ideation intensity and the probabilities of re-contact and referral rose stepwise from *Academic Strain* to *Acute Suicidal Crisis*. Class distributions were similar across study periods.

**Conclusion:**

Adolescent hotline callers can be understood through four recurring profiles that are visible in routine records and meaningfully predict near-term risk and service actions. This typology offers a practical scaffold for triage: prioritize intensive safety planning for crisis presentations; pair social and follow-up supports with peer-related distress; and scale problem-solving and coping support for academic strain.

## 1 Introduction

Adolescence is a period of rapid change, when everyday stressors can escalate quickly into crises. Worldwide, suicide is among the leading causes of death in this age group, and improving access to timely support remains a public health priority (1, 2). Against this backdrop, low-threshold routes to help, such as psychological hotlines, play an important role in the broader mental-health system. Help-seeking in youth rarely follows a straight path. Many adolescents first turn to informal sources (friends, family, teachers) before contacting professionals, and a sizable share never reach formal services (3, 4). Well-documented barriers include stigma, low mental-health literacy, confidentiality concerns, and the belief that problems should be handled alone (5, 6). These obstacles are not uniform. Academic pressure is a particularly salient stressor in East Asian settings, where examination systems shape daily life and family expectations (7). As a result, presentations to frontline services can look quite different even when overall distress is comparable.

Crisis hotlines are designed to meet young people where they are quickly, anonymously, and without costs. Studies suggest that such services can reduce callers’ distress in the moment and provide a bridge to follow-up care (8, 9). The service landscape is also changing: text-based platforms and other digital channels now supplement voice calls, expanding access and reshaping how rapport and risk are assessed (10–12). For services operating at scale, the practical challenge is not only to respond rapidly but also to recognize the varied “faces” of adolescent distress and tailor the response accordingly. Traditional variable-centered analyses, which average across individuals, can blur meaningful differences between subgroups. Person-centered approaches such as latent class analysis (LCA) take a different tack by grouping individuals with similar response patterns and then relating these groups to predictors and outcomes (13–15). In the hotline context, this perspective is useful for two reasons. First, it can reveal distinct constellations of problems that are not obvious from single indicators (for example, academic strain with preserved hope versus multi-domain conflict with hopelessness). Second, it provides an empirical basis for triage and follow-up by linking classes to near-term risk and service use.

The present study applies LCA to a large set of adolescent hotline contacts from a municipal psychological support service. We use indicators that are routinely available in call records, presenting-problem domains and counselor ratings of hope, psychological pain, and suicidal ideation, retaining their ordered structure to avoid loss of information (16–18). Our aims were threefold. First, to identify recurring profiles of help-seeking among adolescents who contact the hotline. Second, to examine how caller characteristics and call context relate to those profiles. Third, to evaluate whether the profiles predict near-term suicidal ideation, re-contact with the service, and referral or triage actions. By grounding the analysis in everyday practice, we seek to provide a framework that hotline teams can use to recognize patterns quickly and to guide responses that are both timely and proportionate.

## 2 Methods

### 2.1 Participants and procedure

We analyzed de-identified case records from the Dongguan “Zhiyin Guanjia Psychological Support Hotline” covering January 1, 2020 to December 31, 2024. The working database was pre-restricted to adolescents aged 10–19 years, so no age-based exclusions were applied at this stage. Eligibility required that the contact concerned the caller’s own difficulties and that core fields were available for analysis, namely demographics, counselor ratings of hope, psychological pain, and suicidal ideation (each recorded on ordered scales), and coded presenting-problem domains.

**Figure 1:**
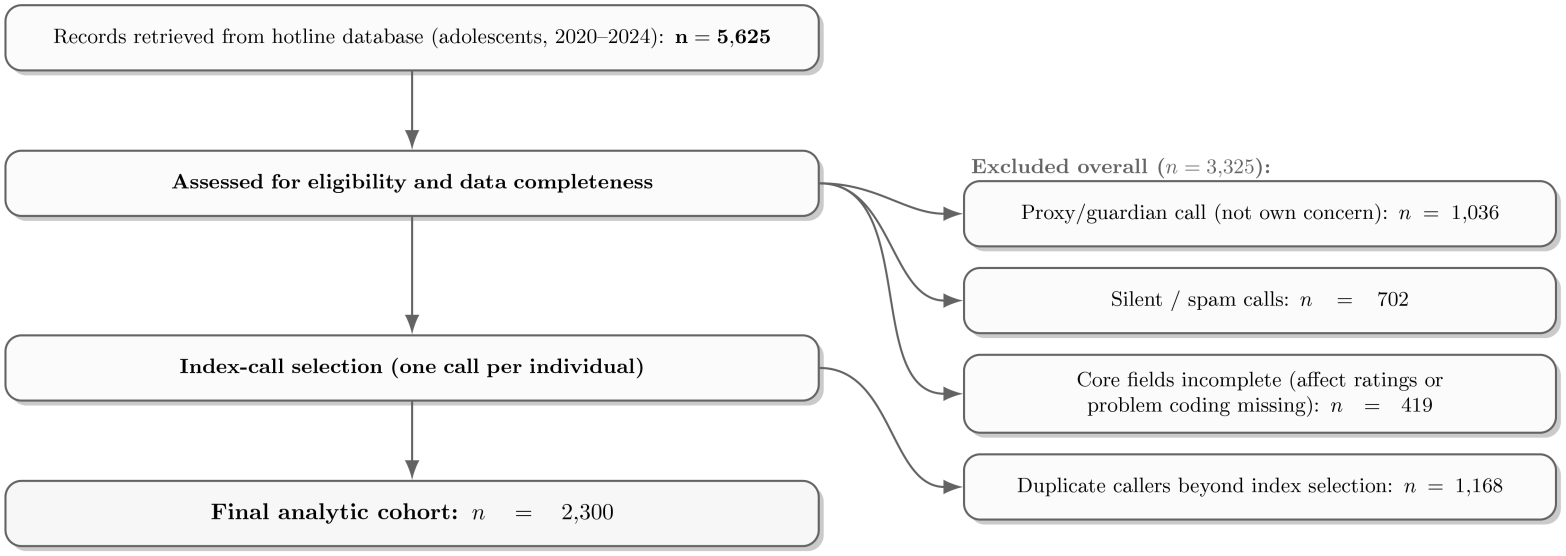
Sample flow from initial adolescent records to the final analytic cohort.

To ensure data quality and one observation per adolescent, we removed (i) proxy/guardian calls (not the adolescent’s own concern), (ii) silent or spam contacts, (iii) records with core fields incomplete, and (iv) duplicate records beyond the selection of a single index call per individual. Calls were linked using pseudonymized keys and counselor notes; the index call was the earliest that met inclusion criteria, or a later call if it contained materially more complete information. For context only, we flagged whether the index call followed a prior contact within 90 days and summarized this as “Contact status” in **Table 1**. Post-index outcomes were evaluated within prespecified windows: near-term suicidal-ideation intensity (clinical monitoring; short window) and, within 30 days, whether the caller re-contacted the hotline and whether a referral/triage action was documented. **Figure 1** shows the flow from the initial retrieval (*n* = 5,625 adolescent records) to the final analytic cohort (*N* = 2,300).

**Table 1:** Descriptive statistics of the participants (Total N = 2,300).

Characteristics	Total (N = 2,300), *n* (%)
Sociodemographics	
Gender	
Male	734 (31.9%)
Female	1,566 (68.1%)
Age (years)	
10–13	603 (26.2%)
14–16	1,172 (51.0%)
17–19	525 (22.8%)
School stage	
Primary	140 (6.1%)
Junior high	1,281 (55.7%)
Senior/Vocational	879 (38.2%)
Household registration	
Urban	1,337 (58.1%)
Rural	963 (41.9%)
Call context	
Time of call	
Daytime	1,469 (63.9%)
Nighttime	831 (36.1%)
Contact status	
First contact	1,796 (78.1%)
Repeat within 90 days	504 (21.9%)
Channel	
Phone	2,111 (91.8%)
Text/Chat	189 (8.2%)
Period	
Pandemic	927 (40.3%)
Post-pandemic	1,373 (59.7%)
Call duration (minutes)	
*<*10	683 (29.7%)
10–19	809 (35.2%)
20–29	471 (20.5%)
≥30	337 (14.7%)
Presenting problems	
Family problems	
Yes	1,032 (44.9%)
No	1,268 (55.1%)
Non-family interpersonal	
Yes	703 (30.6%)
No	1,597 (69.4%)
Study/academic problems	
Yes	821 (35.7%)
No	1,479 (64.3%)
Affective and risk ratings	
Hope (0–4)	
0–1 (low hope)	652 (28.4%)
2 (moderate)	909 (39.5%)
3–4 (high hope)	739 (32.1%)
Psychological pain (0–4)	
0–1 (low)	580 (25.2%)
2 (moderate)	871 (37.9%)
3–4 (high)	849 (36.9%)
Wish-to-die/suicidal ideation (0–3)	
0 (absent)	1,261 (54.8%)
1 (passive)	649 (28.2%)
2–3 (active)	390 (17.0%)
Clinical history	
Prior psychiatric diagnosis	
Yes	329 (14.3%)
No	1,971 (85.7%)
Past self-harm attempt	
Yes	259 (11.3%)
No	2,041 (88.7%)
Service use	
Referral/triage provided	
Yes	559 (24.3%)
No	1,741 (75.7%)
Re-contact within 30 days	
Yes	407 (17.7%)
No	1,893 (82.3%)

### 2.2 Measures

All measures followed routine documentation in the hotline case system and were specified before analysis. We retained the original response formats for Likert-type items to avoid information loss and treated them as ordered categories in subsequent models, consistent with current guidance on ordinal measurement and analysis (16, 19). In particular, we avoided dichotomizing ordered or continuous information, given the well-documented loss of statistical information and interpretability that categorization entails( 17, 18, 20). Counselors documented three affective and risk–relevant judgments for each call: hope, psychological pain, and wish–to–die/suicidal ideation. As described in the record system, each was recorded on an ordered Likert scale used in routine practice (e.g., 0–4); higher values indicated higher hopefulness for hope, and greater intensity for pain and ideation (16, 19).

As part of their induction and ongoing supervision, all counselors receive standardized training on the application of these scales. This is supplemented by group sessions, where counselors review shared case vignettes together to promote and maintain inter-rater consistency.

Reading counselors’ notes, we approached each call as a brief narrative rather than a checklist. Three themes surfaced again and again in adolescent help-seeking, family problems, non-family interpersonal/peer difficulties, and study/academic concerns, consistent with prior work (5, 21, 22). For each call we asked, in turn, whether the conversation touched on each of these themes; whenever it did, we simply marked that theme as present. We did not force a single “main reason”: multiple themes could appear in the same call, reflecting how school pressure, peer conflict, and family tension often intertwine in real life. These yes/no markings gave a clear, lightweight picture of what was discussed and served as the three indicators for the latent class analysis. We also recorded caller characteristics that were relevant for interpretation but were not used to define the latent groups (sex, school stage, prior psychiatric diagnosis, time of day, and a period indicator distinguishing “pandemic” *vs*. “post–pandemic” based on local policy dates). These variables served as external covariates in the three–step models relating characteristics to class membership.

### 2.3 Statistical analysis

Our statistical analysis followed a sequence of four prespecified steps, moving from a broad description of the sample to a detailed validation of the final class solution.

Step 1: Describing the caller population. To set the stage, we first painted a detailed portrait of the adolescents who turn to the hotline for support. We summarized their demographic, clinical, and call-related characteristics to ground the subsequent analyses in the concrete realities of our sample. For clarity and consistency in presentation, all variables were summarized as counts and percentages. This involved grouping naturally ordered variables, such as age, call duration, and the affect/risk ratings, into meaningful categories. This initial descriptive work, summarized in **Table 1**, provided the essential context for interpreting the emergent classes.

Step 2: Searching for underlying patterns. The heart of our study was to discover whether distinct, unobserved subgroups of callers existed within our data. To uncover these patterns, we turned to person-centered mixture modeling (23). We built a series of models based on the core information from each call: the presenting problems (coded as present/absent) and the counselor’s ratings of hope, psychological pain, and suicidal ideation (treated as ordered categories). To ensure we found the most stable and replicable solution, we systematically fit candidate models with one through six potential classes, using a robust maximum likelihood estimator and numerous random starting values to avoid settling on a suboptimal solution (13). The fit statistics for all tested models are presented in **Table 2**.

**Table 2:** Model fit comparison for latent class solutions.

Classes	AIC	BIC	aBIC	Entropy	LMR *p*	BLRT *p*
1	69,025.3	69,380.5	69,145.1	–	–	–
2	66,240.9	66,870.3	66,475.7	0.82	<0.001	<0.001
3	64,935.2	65,838.9	65,285.1	0.85	<0.001	<0.001
4	**64,290.7**	**65,468.6**	**64,655.7**	**0.83**	**0.018**	<0.001
5	64,175.4	65,627.6	64,655.4	0.81	0.118	0.002
6	64,120.1	65,846.6	64,615.2	0.80	0.307	0.071

Bold indicates the selected solution.

Step 3:Selecting the optimal class solution. With several candidate models on the table, the next task was to select the one that best told the story in the data. Our choice was guided by a balance of statistical evidence and real-world clinical meaning, following established guidance (13, 14, 24).

On the statistical side, we consulted a suite of standard fit indices, including the Akaike’s Information Criterion (AIC), the Bayesian Information Criterion (BIC), and the sample-size adjusted BIC (aBIC), alongside likelihood-ratio tests (specifically, the Vuong–Lo–Mendell–Rubin likelihood-ratio test (VLMR) and the Bootstrap Likelihood-Ratio Test (BLRT)) designed to compare nested models (13, 14).

**Figure 2:**
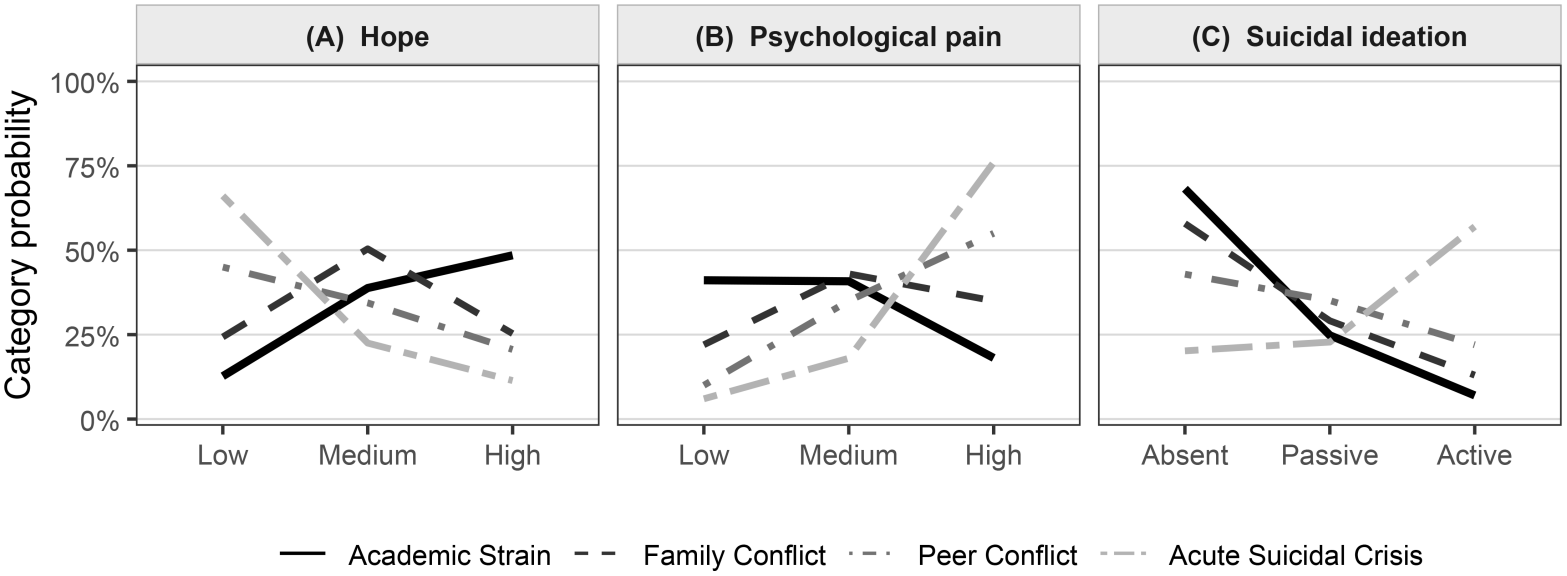
**(A)** Hope, **(B)** Psychological pain, **(C)** Suicidal ideation.

We also considered the entropy, a measure of how confidently individuals are assigned to their most likely class (24). Just as importantly, we weighed these statistics against the interpretability of the solution. We gave preference to a model that not only fit the data well but also produced classes that were large enough to be meaningful (the smallest being 9.9% of the sample) and represented coherent, distinct patterns of distress that could plausibly inform real-world hotline triage. Based on these criteria, the four-class solution was selected for further interpretation, as detailed in **Table 3** and visualized in **Figure 2**.

**Table 3:** Defining characteristics of the four latent classes.

Variables	Class 1 *Academic Strain*	Class 2 *Family Conflict*	Class 3 *Peer Conflict*	Class 4 *Acute Suicidal Crisis*
Class prevalence, *n* (%)	897 (39.2%)	667 (28.8%)	506 (22.1%)	230 (9.9%)
Presenting problems^a^				
Family problems (Yes)	30.1%	71.2%	35.6%	46.8%
Non-family interpersonal (Yes)	18.0%	22.0%	59.8%	40.2%
Study/academic (Yes)	52.6%	21.1%	26.4%	33.3%
Affective and risk ratings^b^				
Hope				
High	48.5%	25.3%	20.6%	11.4%
Medium	38.8%	50.4%	34.4%	22.5%
Low	12.7%	24.3%	45.0%	66.1%
Psychological pain				
Low	41.1%	22.0%	10.0%	6.0%
Medium	40.8%	43.0%	35.0%	18.0%
High	18.1%	35.0%	55.0%	76.0%
Suicidal ideation				
Absent	68.2%	57.9%	42.9%	20.2%
Passive	24.8%	29.1%	35.1%	22.8%
Active	7.0%	13.0%	22.0%	57.0%

Values are conditional probabilities within each class (%). For ‘Presenting problems’, this is the probability of the problem being present. For ordinal scales, values show the percentage distribution across the three levels of the scale.

a These items were the *indicator variables* for the latent class model.

b Hope: 0–1=Low, 2=Medium, 3–4=High; Psychological pain: 0–1=Low, 2=Medium, 3–4=High; Suicidal ideation: 0=Absent, 1=Passive, 2–3=Active.

Step 4:Profiling the classes and testing their predictive power. Once the four-class solution was established, our final step was to bring these abstract groups to life and test their real-world relevance. This involved answering two questions. First, who are the adolescents in these different classes? To explore this, we introduced a comprehensive set of external characteristics (covariates), using a modern three-step approach that corrects for classification uncertainty (15, 25). This allowed us to build the detailed demographic and clinical profiles for each class shown in **Table 4**. Second, does belonging to a particular class predict what happens next? To test this crucial aspect of external validity, we compared the classes on three key outcomes that were not used to create them: near-term suicidal ideation, re-contacting the hotline, and receiving a referral.

**Table 4:** Predictors of class membership from misclassification-corrected three-step models.

Covariates (ref)	Class 2 *vs* 1	Class 3 *vs* 1	Class 4 *vs* 1
Sociodemographics			
Female (Male)	1.34 (1.12, 1.60)^**^	0.92 (0.77, 1.09)	1.58 (1.17, 2.14)^**^
Junior high (Primary)	1.06 (0.75, 1.50)	1.52 (1.05, 2.19)^**^	0.98 (0.62, 1.55)
Senior/Vocational (Primary)	0.87 (0.60, 1.28)	2.10 (1.41, 3.14)^**^	1.10 (0.69, 1.76)
Urban (Rural)	1.05 (0.89, 1.24)	0.96 (0.80, 1.15)	0.90 (0.68, 1.19)
Clinical history			
Prior psychiatric diagnosis: Yes (No)	1.08 (0.84, 1.41)	1.10 (0.85, 1.44)	2.18 (1.56, 3.04)^***^
Past self-harm attempt: Yes (No)	1.12 (0.85, 1.48)	1.18 (0.89, 1.58)	2.70 (1.94, 3.76)^***^
Call context			
Nighttime call: Yes (No)	1.29 (1.07, 1.56)**	1.04 (0.85, 1.28)	1.12 (0.84, 1.49)
Repeat within 90 days (First contact)	1.09 (0.89, 1.33)	0.96 (0.77, 1.19)	1.06 (0.78, 1.44)
Text/Chat (Phone)	1.07 (0.80, 1.44)	1.11 (0.83, 1.50)	0.88 (0.58, 1.32)
Call duration 10–19 min (<10)	1.05 (0.86, 1.28)	0.96 (0.78, 1.18)	1.18 (0.86, 1.62)
Call duration 20–29 min (<10)	1.09 (0.86, 1.38)	1.02 (0.79, 1.32)	1.46 (1.01, 2.11)**
Call duration ≥30 min (<10)	1.12 (0.85, 1.49)	1.06 (0.79, 1.41)	1.82 (1.20, 2.77)**
Period			
Post-pandemic (Pandemic)	1.04 (0.88, 1.22)	0.98 (0.82, 1.18)	0.95 (0.73, 1.24)

Reference class = Class 1 (*Academic Strain*). Values are adjusted odds ratios (95% CI) from misclassification-corrected three-step multinomial models (R3STEP), adjusted for all covariates listed.

Significance:^*^p <.10, ^**^p <.05, ^***^p <.01 (Holm-adjusted). School stage uses *Primary* as the reference; call duration uses <10 minutes as the reference.

We again used state-of-the-art methods that account for classification error, employing the Bolck–Croon–Hagenaars (BCH) weighting for ordered outcomes (15, 26) and the Distal outcome 3-step (DU3STEP) procedure for binary ones, consistent with modern approaches to distal outcome modeling (15, 27).

**Figure 3:**
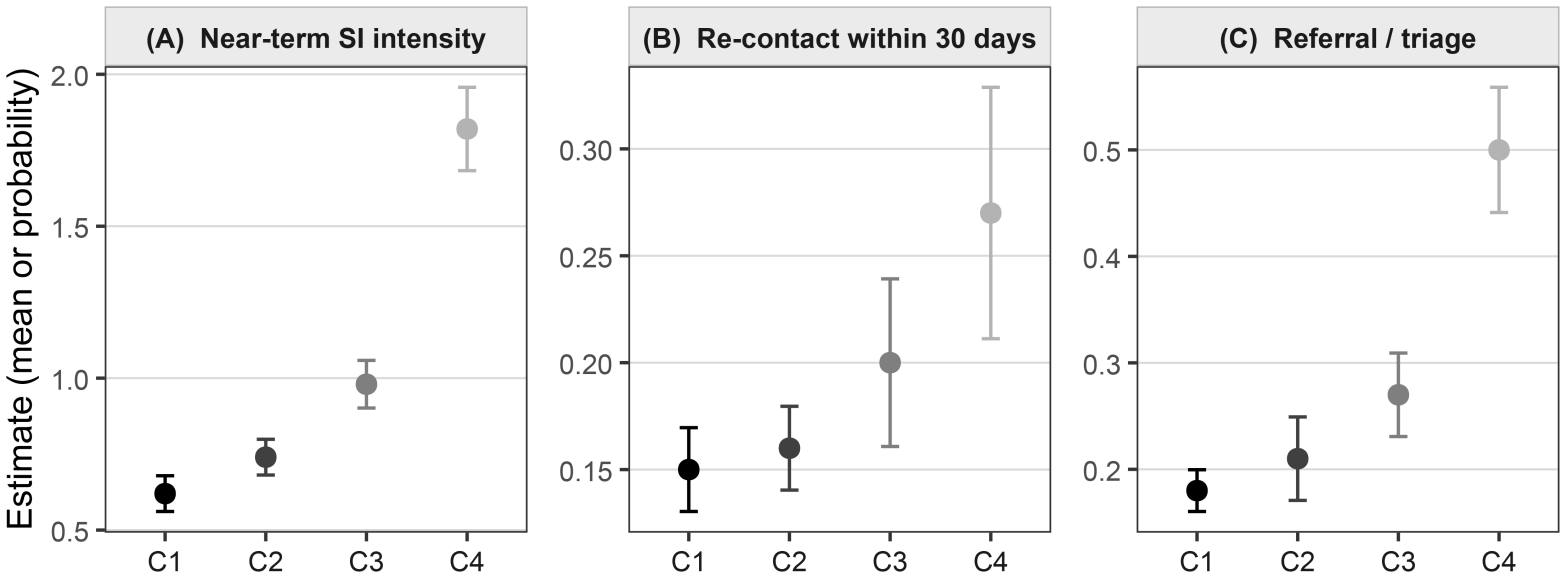
**(A)** Near-term SI intensity, **(B)** Re-contact within 30 days, **(C)** Referral/triage.

To ensure our comparisons were robust, we used an omnibus Wald test for the overall difference across classes, followed by specific pairwise tests with a Holm correction for multiple comparisons (28). The results of these validation analyses are presented in **Table 5** and visualized in **Figure 3**. We also formally tested whether these patterns differed across the pandemic and post-pandemic periods; finding no meaningful differences, we present the more parsimonious results from the pooled sample.

**Table 5:** Distal outcomes by latent class.

Outcome	C1	C2	C3	C4	Omnibus *p*	Pairwise *p*
Near term suicidal ideation intensity^a^	0.62 (0.03)	0.74 (0.03)^*^	0.98 (0.04)^**^	1.82 (0.07)^***^	*<*0.001	C4*>*C1/C2/C3^***^; C3*>*C1^**^; C2*>*C1^*^
Re-contact within 30 days^b^	0.15 (0.01)	0.16 (0.01)	0.20 (0.02)^*^	0.27 (0.03)^**^	0.032	C4*>*C1^**^; C3*>*C1^*^
Referral/triage documented^b^	0.18 (0.01)	0.21 (0.02)	0.27 (0.02)^**^	0.50 (0.03)^***^	*<*0.001	C4*>*C1/C2/C3^***^; C3*>*C1^**^

Cells show class-specific estimates with SE in parentheses. For C2–C4, ^*^/^**^/^***^ denote Holm-adjusted pairwise contrasts *vs* Class 1 (*Academic Strain*): ^*^p <.10, ^**^p <.05, ^***^p <.01.

a Estimated via BCH weighting treating the near-term SI rating as ordered/continuous; larger values indicate higher intensity.

b Estimated via DU3STEP for binary outcomes (logit link), transformed to probabilities for display. “Pairwise *p*” additionally reports non-reference contrasts consistent with the text (Holm-adjusted).

As a guide to interpreting the analyses that follow, our statistical approach can be summarized as a four-step process designed to answer the following key questions in order:

- First (Step 1): Who are the adolescents in our sample? We started by painting a complete descriptive portrait of our 2,300 callers (detailed in **Table 1**).
- Second (Step 2 & 3): Do these callers fall into distinct, “natural” groups based on their patterns of problems and feelings? We used a statistical modeling technique to “listen” to the data. This technique tested whether a 2-group, 3-group, 4-group, 5-group, or 6-group solution best explained the patterns. Based on several fit criteria, we found that a 4-group solution was the most accurate and meaningful way to describe the population. The statistical fit is shown in **Table 2**, and the four resulting profiles (what each group “looks like”) are detailed in **Table 3** and visualized in **Figure 2**.
- Third (Step 4): Now that we have these four groups, are they actually meaningful in the real world? We answered this in two ways: (a) We looked “backward” to see what kinds of callers (e.g., based on gender, age, or clinical history) were most likely to be in each group (**Table 4**). (b) We looked “forward” to see if these groups predicted different, critical outcomes like near-term suicide risk, the likelihood of calling back, or the need for a referral (detailed in **Table 5** and visualized in **Figure 3**).

This full process allowed us to move from a simple description to identifying a robust, validated, and clinically useful typology. This full process allowed us to move from a simple description to identifying a robust, validated, and clinically useful typology.

### 2.4 Ethics statement

This retrospective study was reviewed and approved by the Medical Ethics Committee of Dongguan Seventh People’s Hospital, Guangdong, China (approval No. (2023)008; 19 May 2023). The committee confirmed that analysis of fully anonymized hotline records does not involve intervention and therefore qualifies for a waiver of written informed consent under national regulations and the Declaration of Helsinki. Researchers obtained the data set in de-identified form, with telephone numbers irreversibly hashed before analysis, and no attempt was made to re-identify callers. All procedures complied with the ethical standards for research on human participants promulgated by the National Health Commission of China.

## 3 Results

### 3.1 Descriptive statistics of participants

**Table 1** summarizes the characteristics of the 2,300 adolescents in the analytic cohort. Two thirds were female (1,566; 68.1%). Just over half were aged 14–16 years (1,172; 51.0%), with 26.2% (603) aged 10–13 and 22.8% (525) aged 17–19. Most were enrolled in junior high (1,281; 55.7%) or senior/vocational tracks (879; 38.2%), and a slight majority held urban household registration (1,337; 58.1%).

Regarding the context of contact, nearly two in three calls occurred during daytime hours (1,469; 63.9%). For most adolescents, the index record was a first contact (1,796; 78.1%). Almost all contacts were by phone rather than text/chat (2,111; 91.8%). Calls spanned both the pandemic (927; 40.3%) and post-pandemic periods (1,373; 59.7%). Call lengths varied: 35.2% lasted 10–19 minutes (809), 29.7% were shorter than 10 minutes (683), 20.5% lasted 20–29 minutes (471), and 14.7% were 30 minutes or longer (337). Presenting problems were not mutually exclusive. Family issues were most common (1,032; 44.9%), followed by study/academic concerns (821; 35.7%) and non-family interpersonal difficulties (703; 30.6%). Counselors’ ratings captured a broad range of affect and risk. Hope was typically moderate (909; 39.5%) or high (739; 32.1%). Psychological pain skewed toward moderate (871; 37.9%) and high (849; 36.9%). Suicidal ideation was absent in just over half of calls (1,261; 54.8%), passive in 28.2% (649), and active in 17.0% (390).

Clinical history markers were less frequent but notable: 14.3% (329) had a prior psychiatric diagnosis and 11.3% (259) had a past self-harm attempt. In terms of service actions and near-term engagement, referrals or triage were recorded for 24.3% (559), and 17.7% (407) re-contacted the hotline within 30 days. Overall, these patterns portray a heterogeneous caller population spanning academic strain, family and peer difficulties, and varying levels of distress variation that motivates the latent class analyses that follow.

### 3.2 Class enumeration and selection

We estimated a sequence of latent class models from one to six classes, using many random starts to guard against local solutions. **Table 2** presents the fit statistics and classification quality for each candidate. As expected, AIC declined monotonically with additional classes, while BIC and aBIC showed clearer turning points.

The four–class model offered the best balance of fit and parsimony. It achieved the lowest BIC (65,468.6) and a competitive aBIC (64,655.7), alongside good classification certainty (entropy=0.83). Likelihood–ratio tests supported moving from three to four classes: the LMR test was significant (*p* = 0.018), and the BLRT strongly favored the four–class solution (*p <* 0.001). In contrast, adding a fifth class yielded only marginal improvements on information criteria (a small aBIC decrease to 64,655.4) and a non–significant LMR test (*p* = 0.118), with entropy declining to 0.81. The six–class model continued this pattern (LMR *p* = 0.307; entropy=0.80), suggesting limited incremental structure beyond four classes.

Model interpretability pointed in the same direction. Relative to the three–class model, the four–class specification separated a distinct, higher–risk group without fragmenting the remaining profiles, and did so with adequate class sizes (smallest class: 9.9% of the sample). By contrast, the five– and six–class solutions tended to split existing patterns rather than reveal clearly new profiles, while offering weaker statistical support. Based on the information criteria, LMR/BLRT results, entropy, and substantive clarity, we therefore retained the four–class solution for subsequent profiling and validation.

### 3.3 Class characteristics and naming

We labeled the four classes by combining their most common presenting problems with the joint pattern of hope, psychological pain, and suicidal ideation shown in **Figure 2** and detailed in **Table 3**. The labels are descriptive and intended to aid interpretation rather than to assign diagnoses.

Class 1: *Academic Strain* (*n* = 897; 39.2%). This largest group was defined by study/academic concerns (52.6%), with comparatively fewer family (30.1%) or peer issues (18.0%). The affective profile was the most favorable: nearly half reported high hope (48.5%), psychological pain was mostly low or medium (41.1% and 40.8%), and suicidal ideation was usually absent (68.2%), with a small active fraction (7.0%). The trajectory across the ordered categories in **Figure 2** reflects this comparatively low-risk presentation.

Class 2: *Family Conflict* (*n* = 667; 28.8%). Family problems were the defining feature (71.2%), while academic and peer issues were less prominent (21.1% and 22.0%). Hope clustered at medium (50.4%) with fewer high-hope ratings (25.3%). Psychological pain spread across medium (43.0%) and high (35.0%), and active suicidal ideation appeared in 13.0% of cases. Overall, this class paired clear family strain with moderate distress and intermediate risk.

Class 3: *Peer Conflict* (*n* = 506; 22.1%). Interpersonal difficulties outside the family dominated (59.8%), with a mixed backdrop of family (35.6%) and academic (26.4%) concerns. The affective pattern shifted toward distress: low hope was common (45.0%), high psychological pain was frequent (55.0%), and suicidal ideation rose to 22.0% for the active category. Compared with Class 2, this profile leaned more strongly toward high pain and elevated risk signals.

Class 4: *Acute Suicidal Crisis* (*n* = 230; 9.9%). Although presenting problems were heterogeneous (family 46.8%, peer 40.2%, academic 33.3%), the defining feature was the risk profile. Two thirds reported low hope (66.1%), three quarters had high psychological pain (76.0%), and more than half endorsed active suicidal ideation (57.0%). The steep gradients in **Figure 2** capture this concentration of acute risk.

These four classes map onto recognizable patterns in hotline work: a large group centered on school pressure with low immediate risk; a family-conflict group with moderate distress; a peer-conflict group with higher pain and ideation; and a smaller crisis group marked by very low hope, severe pain, and active suicidal thoughts. These distinctions provide a practical scaffold for the validation analyses that follow.

### 3.4 Covariates predicting class membership

We examined whether caller characteristics and call context were associated with latent class membership using misclassification–corrected three–step models, with *Academic Strain* (Class 1) as the reference (**Table 4**). Odds ratios are adjusted for all listed covariates.

Sociodemographics. Female callers were more likely than males to fall into *Family Conflict* (Class 2 *vs* 1: OR = 1.34, 95%CI: 1.12–1.60) and the *Acute Suicidal Crisis* group (Class 4 *vs* 1: OR = 1.58, 1.17–2.14), with no difference for *Peer Conflict* (Class 3). School stage showed a clear gradient toward the peer–conflict profile: compared with primary school, junior high (OR = 1.52, 1.05–2.19) and especially senior/vocational tracks (OR = 2.10, 1.41–3.14) were associated with Class 3. Urban versus rural registration showed no meaningful differences across classes.

Clinical history. Markers of prior mental–health burden concentrated in the crisis group. A prior psychiatric diagnosis was associated with higher odds of *Acute Suicidal Crisis* (OR = 2.18, 1.56–3.04) but not with Classes 2 or 3. Likewise, a past self–harm attempt strongly predicted Class 4 (OR = 2.70, 1.94–3.76) and was not differentiating for the other contrasts. These patterns align with the sharp risk profile seen for Class 4 in **Figure 2**.

Call context. Nighttime contacts were modestly more likely to belong to *Family Conflict* (OR = 1.29, 1.07–1.56), with no clear differences for the other classes. Call length differentiated the crisis group in a dose–responsive manner: relative to brief calls (*<* 10 minutes), durations of 20–29 minutes (OR = 1.46, 1.01–2.11) and ≥30 minutes (OR = 1.82, 1.20–2.77) were associated with Class 4. Repeated contact within 90 days and channel (text/chat *vs* phone) were not discriminating.

Period. The distribution of classes was similar across the pandemic and post–pandemic periods, with odds ratios close to unity for all contrasts.

These covariate patterns mirror the substantive labels: the *Peer Conflict* group is more common at later school stages; *Family Conflict* shows a slight shift toward females and nighttime calls; and the *Acute Suicidal Crisis* group stands out for prior psychiatric history, past self–harm, and longer calls.

### 3.5 Distal outcomes by class

We next examined whether the classes differed on outcomes not used to define them, using BCH for the ordered near–term suicidal–ideation (SI) rating and DU3STEP for the two binary outcomes (**Table 5**, **Figure 3**).

Near–term suicidal ideation. A clear gradient emerged across classes (omnibus *p <* 0.001). The *Academic Strain* group (C1) showed the lowest mean SI intensity (0.62, SE = 0.03). Estimates rose through *Family Conflict* (C2: 0.74, 0.03) and *Peer Conflict* (C3: 0.98, 0.04), and peaked in *Acute Suicidal Crisis* (C4: 1.82, 0.07). Holm–adjusted contrasts indicated that C4 exceeded all other classes (*p <* 0.01), C3 exceeded C1 (*p <* 0.05), and C2 was modestly higher than C1 (*p <* 0.10). The pattern echoes the risk profiles in **Figure 2**.

Re–contact within 30 days. Re–engagement with the hotline was more common in the classes with greater distress (omnibus *p* = 0.032). Predicted probabilities were 0.15 (0.01) for C1, 0.16 (0.01) for C2, 0.20 (0.02) for C3, and 0.27 (0.03) for C4. Pairwise tests showed higher re–contact for C4 *vs* C1 (*p <* 0.05) and for C3 *vs* C1 (*p <* 0.10), with no detectable difference for C2.

Referral/triage documented. Service actions followed a similarly ordered pattern (omnibus *p <* 0.001). The probability of a recorded referral rose from 0.18 (0.01) in C1 to 0.21 (0.02) in C2 and 0.27 (0.02) in C3, reaching 0.50 (0.03) in C4. C4 exceeded all other classes (*p <* 0.01), and C3 was higher than C1 (*p <* 0.05). This alignment between class profiles and triage decisions supports the practical relevance of the solution.

Overall, the distal results validate the classes in ways that matter for hotline practice: adolescents in the crisis group show markedly higher immediate risk and are more likely to re–contact and receive referrals; the peer–conflict group occupies a middle ground; and the academic–strain group presents with the lowest near–term risk. These are associations rather than causal effects, but they are consistent in direction and magnitude across outcomes, with uncertainty reflected in the confidence intervals plotted in **Figure 3**.

## 4 Discussion

When an adolescent reaches out to a psychological support hotline, they are not just another number; they bring with them a unique story of distress. Our research shows that these individual stories often fall into one of four common, recognizable patterns. We identified a large group of callers primarily struggling with the pressures of *Academic Strain*; two other significant groups caught in either *Family Conflict* or *Peer Conflict*; and a smaller, highly vulnerable group facing an *Acute Suicidal Crisis*. These groups were not just different in the problems they reported. In a key finding, they also showed a clear, step-by-step increase in risk, which was visible in their near-term suicidal thoughts, their likelihood of calling back for more support, and their need for a clinical referral. In the discussion that follows, we’ll explore what these findings mean in the context of other research, how they can inform frontline hotline work, and some of the important details of our study’s methods.

### 4.1 Principal findings in context

First, the mix of academic, family, and peer problems we found reflects the most common reasons young people seek help, both in China and around the world. School pressure is a major trigger for distress during adolescence and has consistently been linked to poorer mental health and self-harm, especially in competitive school systems (29, 30). Likewise, family conflict is a well-known risk factor for suicidal thoughts in young people (31, 32), and difficulties with peers, including bullying and social exclusion, are powerful predictors of elevated risk (33, 34). Our four-class model aligns with these three classic challenges of growing up, while also successfully identifying a separate crisis profile marked by a profound loss of hope, severe psychological pain, and active thoughts of suicide.

Second, the predictable, graded pattern of outcomes gives us strong confidence in the validity of these groups. The intensity of near-term suicidal thoughts rose in a steady, step-by-step pattern from the *Academic Strain* group up to the *Acute Suicidal Crisis* group. The chance of a caller re-contacting the hotline or receiving a referral followed the exact same order. This finding echoes previous studies of crisis services, which show that staff are more likely to activate extra support when a caller is in more acute distress (9, 21, 35). At the same time, our data serve as an important reminder: a significant number of adolescents in the non-crisis classes still reached out again or were referred. This underscores that a caller who seems lower-risk initially may still be on a difficult path and requires careful attention.

Third, the personal characteristics of the callers lined up with the labels we gave each group. The *Peer Conflict* profile was more common among older students in later school stages, a time when social networks and academic stakes become more intense. Nighttime calls were slightly more likely to be about *Family Conflict*, and the crisis group had a concentration of callers with a prior psychiatric diagnosis or history of self-harm. These signals are consistent with broader evidence on how adolescents seek help, including the tendency for girls to reach out more often than boys and to report more internalizing symptoms like anxiety and depression (36, 37). Importantly, despite widespread concern about youth mental health during the COVID-19 pandemic 37, 38), we did not find any significant difference in the distribution of these classes between the pandemic and post-pandemic periods. This doesn’t contradict studies that found an increased overall *demand* for youth mental health services; rather, it suggests that for the adolescents who reached this specific hotline, the *types* of problems and the patterns of risk they presented with remained relatively stable over time.

Our sample was also predominantly female (68.1%), a pattern consistent with extensive literature showing that, across settings, adolescent girls are more likely than boys to recognize distress and seek formal or informal help, especially for internalizing symptoms (4, 5, 39, 40). In our covariate models, females had higher odds of being in the *Family Conflict* and *Acute Suicidal Crisis* classes (*vs*. *Academic Strain*). This imbalance has two implications. First, it cautions that our four-class typology may align more closely with female-typical help-seeking presentations. Second, some male-typical expressions of distress (e.g., externalizing, irritability anger, risk-taking) may be underrepresented among hotline users and, consequently, less visible in our class structure (39, 40).

### 4.2 Implications for hotline practice

These four profiles can serve as a practical guide or framework for hotline staff during triage and follow-up. Adolescents in *Acute Suicidal Crisis* showed the highest immediate risk and were the most likely to be referred, which aligns perfectly with best practices for crisis lines that emphasize immediate safety planning and warm handoffs to other services (9, 35). For the *Peer Conflict* group, their higher levels of psychological pain and suicidal ideation, along with their tendency to call back, suggest that structured follow-up calls and connections to school-based or community programs targeting social skills could be highly valuable. For the *Family Conflict* group, paying attention to the timing of calls (e.g., in the evening), involving family members where appropriate, and using brief, problem-solving techniques may be especially helpful. Finally, although the *Academic Strain* group is the lowest in risk, it is the largest by volume and still contains a significant fraction of youth with passive suicidal thoughts. Offering supports that can be delivered widely, such as psychoeducation, coping skills for managing school stress, and referrals to counseling, could prevent their distress from escalating (9, 29, 36, 36).

Two other practical points emerged. First, call duration was linked to crisis likelihood in a clear pattern: the longer the call, the higher the chance it involved a crisis. While call length is no substitute for a thorough risk assessment, recognizing early that a conversation is becoming long and complex can help supervisors manage their team’s resources in real time. Second, our data highlight the enduring importance of the telephone. While digital channels like text and chat are crucial for expanding access for some young people (33), the phone was still the dominant way adolescents connected with this service. Telephone assessment continues to offer advantages in sensing a caller’s emotional tone, hesitation, and background environment—subtle cues that are vital for a comprehensive risk appraisal (9).

Beyond these individual call-level implications, this typology offers a clear scaffold for broader, system-level service design. First, it provides an evidence-based curriculum for training protocols. New counselors can be trained not just in generic “active listening” but in profile-specific skills, such as role-playing the de-escalation required for the Crisis group versus the scalable psychoeducation needed for the *Academic Strain* group. Second, the findings can directly inform the design of digital triage tools. The simple, routinely-collected indicators used in our model (presenting problems, hope, pain) could be built into intake software to provide a real-time ‘probable profile’ to the counselor, scaffolding (though not replacing) their clinical judgment. Finally, from a policy initiative perspective, the sheer volume of the *Academic Strain* class (39.2%) provides a powerful data point for hotline administrators to advocate for systemic, school-based mental health interventions that address this clear and substantial unmet need.

### 4.3 Methodological considerations

A key decision we made in our analysis was to treat the counselor ratings with care, modeling them as ordered categories rather than simply splitting them into “yes” or “no.” This approach preserves valuable information about the severity of a problem and avoids the well-known issues that come from using arbitrary cut-points, which can reduce statistical power and make results harder to interpret (16–18, 41, 42). To select the best number of classes, we balanced several factors, including statistical fit indices (AIC/BIC/aBIC), significance tests (LMR, BLRT), and the interpretability of the groups, an approach widely supported in the field (13–15, 25). We then used advanced three-step methods that correct for classification error when relating external factors to the classes and when estimating future outcomes (BCH and DU3STEP) (15, 26, 27).

### 4.4 Limitations and future directions

A primary limitation of this study is its single-site design, with data drawn exclusively from one municipal hotline in Dongguan, China. This context should be borne in mind when interpreting the findings. The relatively high prevalence of the *Academic Strain* profile (39.2%) is consistent with the high-stakes educational environment that characterizes many East Asian settings (7). Outside East Asia, national and regional helpline reports show a different mix of presenting concerns. For example, Australia’s national Kids Helpline reports that contacts most commonly relate to *mental health or emotional wellbeing*,

with *family* and *peer relationship* issues also prominent your town (43). European child helplines likewise indicate that *mental health* was among the leading reasons for contact in 2022 Child Helpline International (44, 45). This suggests that while interpersonal stressors are universal, the dominance of *Academic Strain* as the single largest profile in our urban Chinese sample may be context-specific. Generalizability may also be limited by urban–rural differences within China. Cross-sectional evidence from Guangdong shows that rural and rural-to-urban migrant school-age children have poorer mental health status than urban peers (46). At the service level, studies document substantial rural–urban disparities in the utilization of mental health inpatient care in China, with lower use among rural residents (47). Rural adolescents also face distinct stressors linked to parental migration; meta-analytic evidence indicates markedly worse mental health among left-behind children compared with non–left-behind peers (48). Replication in rural and non–East Asian settings is therefore a clear priority to establish what is universal versus context-specific in this typology. Future research should therefore prioritize replicating this typology in rural and non–East Asian settings to establish what is universal versus context-specific.

A second limitation relates to measurement. Preserving the ordinal information does not remove the fundamental constraint that these indicators are, themselves, based on counselor ratings on internal, nonstandardized scales. This approach is inherent to the fast-paced, real-world hotline setting but introduces potential subjectivity and inter-rater variability. Although our staff undergo training and group sessions to promote consistency, these ratings are not a substitute for validated, psychometric instruments. Future research, where feasible, would be strengthened by integrating brief, validated self-report measures. For example, services could explore administering established tools, such as the 4-item Suicidal Behaviors Questionnaire-Revised (SBQ-R) (49) or brief visual analog scales for distress, via an optional, automated text or web link post-call. This would provide a valuable, standardized datapoint to corroborate counselor judgment and track outcomes over time.

A third limitation is inherent to the LCA method itself. As a person-centered model, it assigns individuals to their ‘best-fit’ class, which necessarily simplifies a more complex and overlapping reality. In practice, the boundaries between *Peer Conflict*, *Family Conflict*, and *Academic Strain* are porous; distress in one domain often spills over into others. Our typology provides a practical ‘first-glance’ framework for triage, but it does not capture the dynamic interplay of these co-occurring stressors. Future research could move beyond classification to explore this complexity directly. For instance, a network analysis approach could model how these problems (e.g., family conflict, academic pressure, peer isolation, and suicidal ideation) are interconnected, mutually reinforcing, and identify which stressors are most ‘central’ to the adolescent distress network. To capture overlap and joint structure more directly in future research, several extensions are promising: (i) mixed-membership (“fuzzy”) models that allow partial membership in multiple profiles (50, 51), (ii) factor–mixture models that combine a continuous risk/acuity dimension (e.g., hope–pain–ideation) with discrete content profiles (52, 53), and (iii) network analysis to map how stressors and ideation interconnect and to identify central targets for intervention (54, 55).

A fourth limitation is the reliance on short-term, service-level outcomes. Our 30-day window for recontact and referral serves as a proximal indicator of immediate risk and engagement, but it cannot capture longer-term clinical trajectories. Future studies should, where ethically and logistically feasible, attempt to track adolescents over longer periods (e.g., 6–12 months) to assess sustained impact, such as clinical improvement or desistance from self-harm. Moreover, this quantitative typology only tells part of the story. Future work would be substantially enriched by incorporating qualitative follow-up interviews. This approach is essential to understand the lived experiences and unmet needs of adolescents within each profile, providing crucial context that our current data lacks.

Looking ahead, these findings open up several exciting possibilities. The four profiles could be translated into simple decision-support tools that run in the background of hotline software, highlighting potential risk patterns to assist, but not replace, clinical judgment. Future studies should also prospectively test whether a profile-informed approach to triage, such as offering structured follow-up calls to everyone in the *Peer Conflict* group, improves outcomes and reduces near-term risk. Finally, linking hotline data to school or clinic records, where possible, could offer a much richer understanding of a young person’s journey through the care system after they call for help.

### 4.5 Practical summary for hotline staff

In essence, this study provides a practical field guide for frontline staff. Our findings suggest that adolescent callers often fit one of four common profiles, each benefiting from a tailored response:

For the *Acute Suicidal Crisis* group: The priority is immediate safety. This means moving beyond de-escalation to collaborative safety planning (e.g., “What is one thing that can keep you safe for the next hour?”), activating emergency protocols if the caller is in imminent danger, and ensuring a “warm handoff” or direct referral to local emergency services.

For the *Peer Conflict* group: This group shows high distress and is likely to call back. Staff can respond by proactively offering a structured follow-up call. In these calls, counselors can focus on brief, evidence-based skills like social problem-solving (“Let’s walk through three possible ways to handle that text message”) or emotional regulation techniques (e.g., grounding exercises to use before responding to a peer).

For the *Family Conflict* group: Callers often feel unheard and powerless. The key response is validation of their feelings. This can be paired with practical skills training in assertive communication (e.g., practicing “I-statements” like “I feel [emotion] when [event] happens”) and exploring de-escalation or safety plans for volatile situations at home.

For the *Academic Strain* group: As the largest group, the response needs to be efficient and scalable. This involves in-the-moment psychoeducation (e.g., normalizing their stress and challenging perfectionism) and providing concrete, non-clinical coping tools. Examples include time management strategies, simple relaxation exercises (like box breathing), or cognitive reframing (e.g., “What’s the evidence that failing this one quiz means you’ll fail the year?”).

## 5 Conclusions

Adolescent callers to a psychological support hotline are not a homogeneous group. Our research shows that their individual stories of distress often fall into one of four common patterns: a large group struggling with school pressure, two groups caught in conflicts with family or with peers, and a smaller, high-risk group facing an immediate suicidal crisis. These profiles are far more than just descriptive labels. They act as vital indicators, helping us predict a young person’s immediate suicide risk and the kind of support they will need from counselors. By providing a clear and simple vocabulary to describe these common patterns of distress, this work offers a practical tool for frontline staff and a foundation for building more responsive and effective crisis care for young people.

## Data Availability

The raw data supporting the conclusions of this article will be made available by the authors, without undue reservation.
